# Prediction of NB‐LRR resistance genes based on full‐length sequence homology

**DOI:** 10.1111/tpj.15756

**Published:** 2022-04-18

**Authors:** Giuseppe Andolfo, Juliane C. Dohm, Heinz Himmelbauer

**Affiliations:** ^1^ Department of Agricultural Sciences University of Naples ‘Federico II’ Via Università 100 80055 Portici (Naples) Italy; ^2^ Institute of Computational Biology, Department of Biotechnology University of Natural Resources and Life Sciences Vienna Muthgasse 18 1190 Vienna Austria

**Keywords:** plant breeding, disease resistance genes, genome annotation, repeat masking, gene prediction, *Beta vulgaris*, *Solanum lycopersicum*, *Cucurbita*

## Abstract

The activation of plant immunity is mediated by resistance (R)‐gene receptors, also known as nucleotide‐binding leucine‐rich repeat (NB‐LRR) genes, which in turn trigger the authentic defense response. R‐gene identification is a crucial goal for both classic and modern plant breeding strategies for disease resistance. The conventional method identifies NB‐LRR genes using a protein motif/domain‐based search (PDS) within an automatically predicted gene set of the respective genome assembly. PDS proved to be imprecise since repeat masking prior to automatic genome annotation unwittingly prevented comprehensive NB‐LRR gene detection. Furthermore, R‐genes have diversified in a species‐specific manner, so that NB‐LRR gene identification cannot be universally standardized. Here, we present the full‐length Homology‐based R‐gene Prediction (HRP) method for the comprehensive identification and annotation of a genome's R‐gene repertoire. Our method has substantially addressed the complex genomic organization of tomato (*Solanum lycopersicum*) NB‐LRR gene loci, proving to be more performant than the well‐established RenSeq approach. HRP efficiency was also tested on three differently assembled and annotated *Beta* sp. genomes. Indeed, HRP identified up to 45% more full‐length NB‐LRR genes compared to previous approaches. HRP also turned out to be a more refined strategy for R‐gene allele mining, testified by the identification of hitherto undiscovered *Fom‐2* homologs in five *Cucurbita* sp. genomes. In summary, our high‐performance method for full‐length NB‐LRR gene discovery will propel the identification of novel R‐genes towards development of improved cultivars.

## Significance Statement

*De novo* prediction of plant disease resistance (R)‐genes is difficult due to their intrinsic features. We introduce Homology‐based R‐gene Prediction, a novel strategy for the comprehensive prediction of R‐genes and their alleles, outperforming previous approaches of R‐gene discovery.

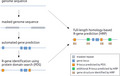

## INTRODUCTION

Plant innate immunity is a complex system to defend against disease and pests. It is based on two pathogen recognition layers, known as pathogen‐associated molecular pattern (PAMP)‐triggered immunity (PTI) and effector‐triggered immunity (ETI) (Jones & Dangl, 2006). In PTI, PAMPs are perceived by receptors at the surface of the cell, but pathogens may be able to arrest PTI signaling through the production of effector proteins that inhibit the activation of defense responses (Andolfo & Ercolano, 2015). The effectors may be recognized by the second defense level involving resistance (R)‐genes that trigger ETI. Therefore, the identification of R‐genes in plant genomes is useful for both classical and innovative strategies of disease resistance breeding (Andolfo et al., 2021a,b; Capistrano‐Gossmann et al., 2017).

The major class of plant R‐genes encodes nucleotide‐binding (NB) and leucine‐rich repeat (LRR) proteins (Michelmore et al., 2013). The origin of the NB‐LRR pairwise domain combination dates back to over 3.5 billion years ago and it represents the R supra‐domain of green plants (Andolfo et al., 2019). The NB domain is the most conserved region, while the C‐terminal LRR domain that provides target specificity is extremely variable. NB‐LRR genes are generally divided into three protein classes (TNL: TIR‐NB‐LRR; CNL: CC‐NB‐LRR; and RNL: RPW8‐NB‐LRR), based on different N‐terminus architecture (Andolfo et al., 2019; Andolfo et al., 2020). The R‐gene defense arsenal of plants is generally composed of partial (single or fragmented domains) and full‐length (NB‐LRR) genes. Although partial genes could be considered pseudogenes, they are often expressed and could have a function in the regulation of full‐length R‐genes (Marone et al., 2013).

Generally, R‐genes are organized in clusters of tandemly duplicated genes, although they can also be singularly dispersed in the genome (Barchi et al., 2019; Di Donato et al., 2017). To date, automatic gene annotation pipelines are incapable to predict and correctly identify NB‐LRR loci, as their genomic organization in gene clusters leads to missing and fragmented annotations (Andolfo et al., 2014; Jupe et al., 2013). The multiplicity of similar sequences can cause local genome assembly collapse and annotation problems (Tørresen et al., 2019). This is compounded by the fact that R‐genes are often expressed at low levels, so RNA sequencing (RNA‐Seq) data would not provide sufficient supporting evidence for gene prediction (McHale et al., 2006). Additionally, the NB‐LRR loci may be masked by genome annotation pipelines using public databases for transposable elements (TEs), as R‐genes are sometimes annotated as being repetitive sequences (Bayer et al., 2018). Therefore, the identification of NB‐LRR genes in unexplored genomic regions is an important goal (Andolfo et al., 2013; Meyers et al., 2003).

Generally, pipelines for NB‐LRR gene identification attempt to detect the domains associated with R‐genes in protein sequences derived from automated gene annotation of assembled genomes (Meyers et al., 2003; Andolfo et al., 2013). Recently, two tools were developed to annotate NB‐LRR genes directly in the genome using conserved motifs/domains of R‐genes (Steuernagel et al., 2020; Toda et al., 2020). However, this type of approach is not always able to correctly solve the complex intron–exon structures of NB‐LRR genes (Toda et al., 2020). Moreover, the use of a singular protein domain or short motifs for R‐gene discovery does not allow the prediction of entire NB‐LRR gene models (Funk et al., 2018). The task to specifically predict the gene structure of full‐length NB‐LRR genes directly within the genomes of studied taxa has not yet been addressed.

Here, we present the full‐length Homology‐based R‐gene Prediction (HRP) pipeline to comprehensively identify full‐length NB‐LRR gene models within genome assemblies. We compared the performance of the HRP method to that of the manually curated tomato (*Solanum lycopersicum*) ‘Resistance gene enrichment and sequencing’ (RenSeq) annotation (Andolfo et al., 2014). Additionally, we tested the HRP method efficiency in overcoming biases due to repeat masking in three *Beta* sp. and five *Cucurbita* sp. genomes for full‐length NB‐LRR gene detection and allele mining, respectively.

## RESULTS

### Full‐length Homology‐based R‐gene Prediction

Once a genome sequence has been assembled, gene prediction pipelines are used to generate a gene set for the respective assembly. Within such a gene set, R‐genes can be detected using protein motif/domain‐based search (PDS). However, automated gene prediction leads to incomplete representation of R‐genes within gene sets (Andolfo et al., 2014; Jupe et al., 2013). We noted that automatically determined gene sets yet contain a sufficiently large number of full‐length R‐gene representatives, which can be taken advantage of to detect further paralogous R‐genes that have escaped annotation. Thus, we introduce a two‐level homology search, firstly using protein domains to identify an initial set of R‐genes in the gene set of the automated gene prediction and secondly using these R‐genes for full‐length homology searches in the genome assembly. With this approach of full‐length HRP we are able to identify additional R‐genes including their gene structure.

To evaluate the performance of the HRP method for NB‐LRR gene prediction within a sequenced genome, we compared the HRP results with RenSeq, known as a high‐quality manual R‐gene annotation method (Jupe et al., 2013). Using RenSeq, 326 NB‐LRR genes were previously identified in the tomato genome (*S. lycopersicum* Heinz‐1706 v2.4) (Andolfo et al., 2014). For comparison, we used 173 tomato full‐length protein sequences (Table S1), containing the NB and LRR domains regardless of other domains as input for the HRP method to predict R‐genes. This dataset had been determined by conventional domain search in an independent study (Andolfo et al., 2013).

HRP identified 363 NB‐LRR genes in the tomato genome (Table 1; Table S2), including 103 of 105 novel genes that had previously been identified by RenSeq (Table S3). Only two partial genes (RDC0002NLR0028 and RDC0002NLR0034) reported by Andolfo et al. (2014) could not be predicted. These genes are transcriptionally inactive pseudogenes, and, most likely, their limited length of 483 and 255 bp, respectively, did not permit the prediction of NB‐LRR domain‐encoding regions. We manually analyzed all six possible reading frames of RDC0002NLR0028 and RDC0002NLR0034 and found on average seven stop codons in these pseudoproteins.

**Table 1:** Number of tomato NB‐LRR genes as identified by HRP, RGAugury and RenSeq in *S. lycopersicum* Heinz‐1706 v2.4

Protein domains^a^	RenSeq^b^	RGAugury^c^	HRP
Full‐length			
CC‐NB‐LRR	193	151	198
RPW8‐NB‐LRR	2	–	2
TIR‐NB‐LRR	26	19	31
Total full‐length	221	170	231
CC	–	–	3
Partial			
CC‐NB	14	8	4
RPW8	–	–	1
TIR‐LRR	1	–	–
TIR‐NB	3	7	5
NB	57	63	64
TIR	10	–	7
LRR	20	2	48
Total partial	102	80	132
Total	326	250	363 (324)^d^

^a^ CC: coiled coil; LRR: leucine‐rich repeat; NB: nucleotide binding; RPW8: resistance to powdery mildew 8; TIR: Toll/interleukin receptor.

^b^ Tomato R‐gene enrichment and sequencing annotation (Andolfo et al., 2014).

^c^ NB‐LRR genes annotated by RGAugury (Li et al., 2016) in *S. lycopersicum* Heinz‐1706 v2.3 proteome.

^d^ Number of confirmed NB‐LRR genes previously identified by the RenSeq approach in brackets.

Surprisingly, the HRP method identified 39 novel NB‐LRR genes in the tomato genome (Table 1), of which 10 were full‐length NB‐LRR genes (five CNLs and five TNLs). No novel RNL gene was identified, but the Activated Disease Resistance 1 (*ADR1*) and N Requirement Gene 1 (*NRG1*) homologs were confirmed (Andolfo et al., 2020). RNL genes, known as ‘helpers’, are involved in immune signaling that is triggered by TNL and CNL genes when detecting pathogen effectors. The remaining 27 novel tomato R‐genes were partial NB‐LRR genes which encode domains similar to plant R‐genes (Table 1).

To evaluate the HRP accuracy in the prediction of gene models, we compared the annotation of the HRP input dataset as released by the international Tomato Annotation Group (iTAG) with the gene structures predicted by HRP. Although only 48 (out of 173) pairs of R‐loci showed an identical intron–exon structure (Table S4), 97% of coding sequences was shared between the 173 gene models annotated by iTAG and predicted by HRP. On average, the 173 full‐length NB‐LRR proteins predicted by HRP (average length 1000 amino acids) were longer than those issued by iTAG (average length 926 amino acids).

**Figure 1:**
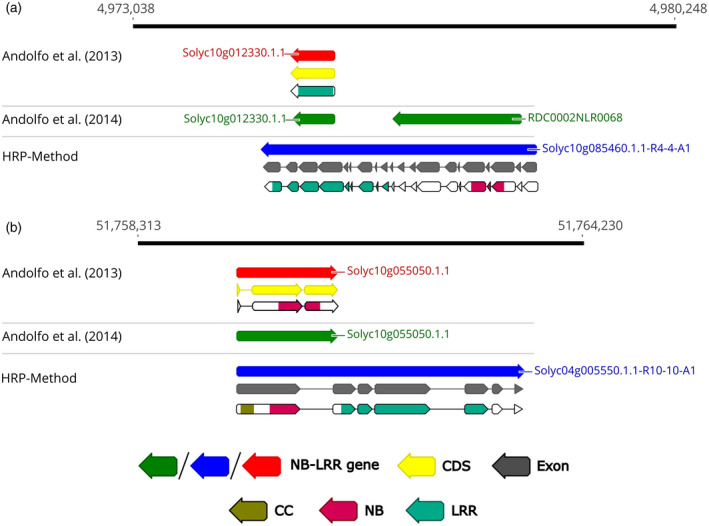
Re‐annotation of two erroneously annotated NB‐LRR genes located on chromosome 10 of the *S. lycopersicum* Heinz‐1706 genome. (a) Mapping of tomato full‐length NB‐LRR gene Solyc10g085460 identified a single gene encompassing two previously (Andolfo et al., 2014) annotated gene models (green arrows). (b) The Solyc10g055050 gene (red arrow) was confirmed by RenSeq as partial gene (green arrow). The corrected exon annotation identified by the HRP method was confirmed in an InterProScan analysis. Domains are shown as gold (CC), rose (NB) and spring green (LRR) arrows. [Colour figure can be viewed at wileyonlinelibrary.com]

In Figure 1, we present two examples of R‐genes wrongly annotated by RenSeq, on tomato chromosome 10, which were re‐annotated as two full‐length NB‐LRR genes by HRP. Firstly, Andolfo et al. (2014) had identified two distinct partial genes (Solyc10g012330.1.1 and RDC0002NLR0068) located at genomic interval 4 973 038–4 980 248, while annotation by HRP suggested a single full‐length NB‐LRR gene (Solyc10g085460.1.1‐R4‐4‐A1) at this locus (Figure 1a). In the second example we show that the previously annotated Solyc10g055050.1 was a partial R‐gene lacking the LRR domain, while HRP resulted in the annotation of the full‐length NB‐LRR gene Solyc04g005550.1.1‐R10‐10‐A1 (Figure 1b).

Finally, we compared the performance of HRP with that of RGAugury, an automated version of the PDS method (Li et al., 2016). Using RGAugury we detected only 250 NB‐LRR genes in the tomato proteome, in contrast to 363 R‐genes detected with HRP (Table 1; Table S5).

### NB‐LRR gene prediction in two differently annotated sugar beet genome assemblies

R‐gene prediction may be incomplete due to different approaches in repeat masking during automated genome annotation. To verify the efficiency of our HRP method in overcoming this bias we analyzed two sugar beet (*Beta vulgaris* ssp. *vulgaris*) genome assemblies, RefBv‐1.0 and RefBeet‐1.2, prepared from the same double‐haploid sugar beet genotype KWS2320. The two assemblies differ with respect to the sequencing technology that was used. The assembly RefBv‐1.0 was generated from Illumina sequencing data, while RefBeet‐1.2 was assembled chiefly based on data from the Roche/454 sequencing platform (Dohm et al., 2014). The initial gene annotation of both assemblies was performed using the Augustus pipeline with *Arabidopsis* parameters. Later on, the annotation of RefBeet‐1.2 was updated, after adapting gene prediction parameters to Caryophyllales. Also, repetitive sequences were excluded from integration into gene models more efficiently (Minoche et al., 2015). Therefore, the updated gene set of RefBeet‐1.2 is expected to be more accurate than the one of RefBv‐1.0. The question was to what extent the HRP method would be able to improve the annotation of R‐genes in the two assemblies.

**Figure 2:**
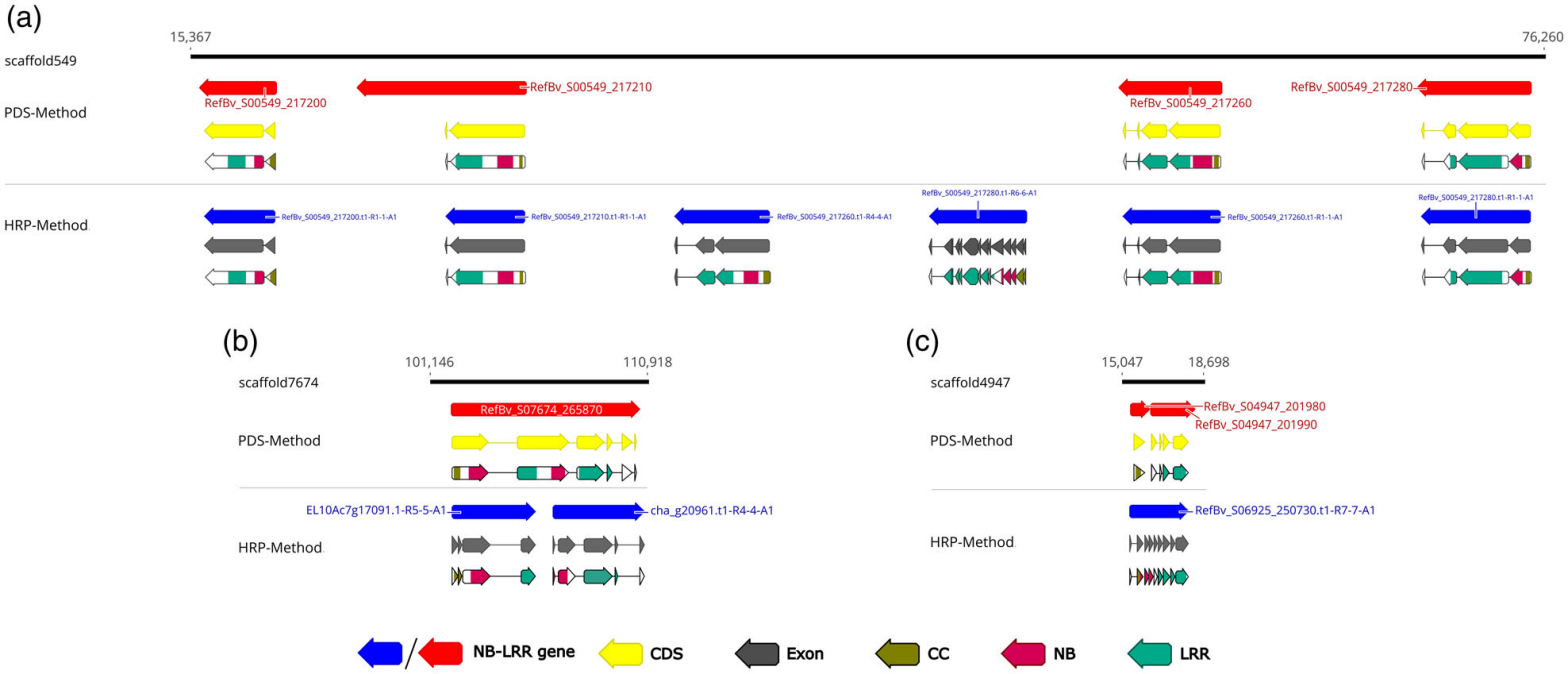
Homology‐based NB‐LRR gene prediction in sugar beet. Detailed analysis (a) of an NB‐LRR gene cluster on RefBv‐1.0 scaffold 549 and re‐annotation of two erroneously (b) fused and (c) split NB‐LRR genes located on RefBv‐1.0 scaffolds 7674 and 4947, respectively. The *Beta vulgaris* genomic regions with annotated R‐genes by PDS (red arrows) and the novel full‐length NB‐LRRs predicted by full‐length Homology‐based R‐gene Prediction (HRP) (blue arrows) are shown. The corrected annotation was confirmed in an InterProScan analysis. Domains are shown as gold (CC), rose (NB) and spring green (LRR) arrows. [Colour figure can be viewed at wileyonlinelibrary.com]

The input sequences for HRP were generated by applying an NB‐LRR PDS on the predicted genes of six sequenced sugar beet genomes, one leaf beet (chard) genome, two wild beet (*B. vulgaris* ssp. *maritima* and *B. patula*) genomes, and one spinach (*Spinacia oleracea*) genome (Table 2). In total, PDS identified 1139 full‐length NB‐LRR genes in these gene sets (Table S4). In the gene sets of RefBeet‐1.2 and RefBv‐1.0 we identified 97 and 146 full‐length NB‐LRR genes, respectively (Table 3), confirming the expectation that different repeat masking approaches led to differing numbers of predicted R‐genes during the automated genome annotation process (Dohm et al., 2014; Minoche et al., 2015). Using the cumulative set of 1139 NB‐LRR genes identified in nine genomes of beets and one spinach genome as input (Table 2; Table S6) the HRP method identified 178 and 172 full‐length NB‐LRR genes in the unmasked assemblies RefBeet‐1.2 and RefBv‐1.0, respectively (Table 3). HRP predicted also 53 (out of 231) and 82 (out of 254) partial NB‐LRR genes in RefBeet‐1.2 and RefBv‐1.0, respectively (Table S7). These numbers are not only much higher but also similar between the assemblies, indicating that repeat masking may be a major reason for missed R‐genes in the initial annotations. Aside from one gene each assigned to TNL and RNL, all other detected NB‐LRR genes were from the CNL class. Using full‐length proteins as search sequences we could resolve the gene locations and gene structures more comprehensively than using PDS. For example, in RefBv‐1.0 PDS did not find all members of a gene cluster and additional genes between them could be found using HRP (Figure 2a), one gene was predicted which turned out to be two separate genes after applying HRP (Figure 2b) and two adjacent predicted genes were actually found to belong to only one gene (Figure 2c).

**Table 2:** Gene sets used to generate the input set of protein sequences for the HRP method and number of full‐length NB‐LRR genes identified by PDS

Species	Accession	Protein‐coding genes	References	R‐genes^b^
*Beta vulgaris* ssp. *vulgaris* var. *altissima*	KWS2320	25 813^a^	Dohm et al., 2014	146
	KWS230/DH1440	25 368	Dohm et al., 2014	138
	STR06A6001	31 355	Dohm et al., 2014	139
	YMoBv	25 927	Dohm et al., 2014	140
	YTiBv	25 626	Dohm et al., 2014	140
	C869/EL10	24 255	Funk et al., 2018	143
*Beta vulgaris* ssp. *vulgaris* var. *cicla*	M4021	27 513	Lehner et al., 2021	76
*Beta vulgaris* ssp. *maritima*	WB42	27 617	Rodríguez del Río et al., (2019)	84
*Beta patula*	BETA548	25 069	Rodríguez del Río et al., (2019)	84
*Spinacia oleracea*	Viroflay	21 703	Minoche et al., 2015	49
			Total	1139

^a^ Number of protein‐coding genes predicted in the RefBv‐1.0 genome assembly.

^b^ Number of full‐length NB‐LRR genes.

**Table 3:** Comparison of NB‐LRR genes identified in two different isogenic *B. vulgaris* ssp. *vulgaris* genome assemblies by R‐protein motif/domain‐based search (PDS) and by full‐length Homology‐based R‐gene Prediction (HRP)

Protein domains^a^	PDS		HRP	
	RefBeet‐1.2	RefBv‐1.0	RefBeet‐1.2	RefBv‐1.0
Full‐length				
CC‐NB‐LRR	95	144	176	170
RPW8‐NB‐LRR	1	1	1	1
TIR‐NB‐LRR	1	1	1	1
Total full‐length	97	146	178	172
Partial				
CC	10	9	2	6
CC‐NB	5	4	1	4
CC‐LRR	14	–	–	–
TIR‐NB	2	2	2	2
NB	30	31	26	29
TIR	2	1	2	1
LRR	43	42	20	40
Total partial	106	89	53	82
Total	203	235	231	254

^a^ CC: coiled coil; LRR: leucine‐rich repeat; NB: nucleotide binding; RPW8: resistance to powdery mildew 8; TIR: Toll/interleukin receptor.

Among the HRP‐predicted R‐genes in the sugar beet reference genotypes, 98% of RefBeet‐1.2 R‐genes had a match to at least one RefBv‐1.0 partial/full‐length R‐gene, while only 92% of RefBv‐1.0 R‐genes were found in the RefBeet‐1.2 R‐gene annotation. We noticed that only 147 (out of 172) full‐length NB‐LRR genes identified in RefBv‐1.0 showed an identical R‐locus in RefBeet‐1.2 (Table S8). No exact match was found for the remaining 25 and 31 CNL genes identified in RefBv‐1.0 and RefBeet‐1.2, respectively. Most likely, this is due to technical issues related to the generation of the two sugar beet genome assemblies, even though they are based on the same double haploid genotype, KWS2320.

### R‐gene identification in a sugar beet genome sequence assembled from third‐generation sequencing data

Recently, an NB domain identification method was developed to discover R‐genes within the EL10.1 sugar beet genome assembly prepared from third‐generation Pacific Biosciences (PacBio) sequencing data (Funk et al., 2018). The authors performed a search for R‐genes based on hidden Markov models (HMMs) which had been developed using 185 NB‐LRR nucleotide sequences previously annotated in the EL10.1 gene set (Funk et al., 2018). We used the HRP method to analyze the PacBio‐based EL10 assembly and to compare the results with the PDS method and the NB‐encoding HMM‐based gene annotation by Funk et al. (2018).

A total of 220 NB‐LRR genes (143 full‐length and 77 partial) were annotated in the EL10.1 genome assembly by PDS (this study) and 231 tentative NB‐like domains were identified by Funk et al. (2018). Surprisingly, using the 143 full‐length sequences found by PDS as input (Table 2), HRP predicted 263 NB‐LRRs (Table S9) of which 37 had not been reported by Funk et al. (2018). The 220 NB‐LRR genes identified by PDS were all confirmed by HRP, while only five of the 231 tentative NB‐like domains (Bv.nbarc.0005, Bv.nbarc.0023, Bv.nbarc.0039, Bv.nbarc.0046 and Bv.nbarc.0083) did not reside in NB‐LRR loci predicted by HRP. The length of these five tentative NB‐like domains ranged from 146 to 310 nucleotides and they were considered as pseudoproteins (Funk et al., 2018). Probably, the five pseudoproteins were too short to encode an NB domain. Indeed, the NB Pfam domain of EL10.1 NB‐LRR genes predicted by HRP is on average 200 amino acids long. Finally, 16 out of 231 tentative ‘NB‐like’ domains (Bv.nbarc.0067, Bv.nbarc.0069, Bv.nbarc.0081, Bv.nbarc.0090, Bv.nbarc.0091, Bv.nbarc.0095, Bv.nbarc.0098, Bv.nbarc.0099, Bv.nbarc.0101, Bv.nbarc.0104, Bv.nbarc.0109, Bv.nbarc.0112, Bv.nbarc.0116, Bv.nbarc.0118, Bv.nbarc.0163 and Bv.nbarc.0179) reported by Funk et al. (2018) overlapped with the LRR domain‐encoding region of partial NB‐LRR genes identified by HRP. Most likely, the HMMs used by Funk et al. included nucleotide regions encoding also the LRR domain.

### R‐gene allele mining using the HRP method within *Cucurbita* sp. genome assemblies

Plant breeding for disease resistance is based on the identification of alleles conferring resistance. Allele mining is a high‐throughput strategy to isolate genetic variants of novel R‐genes (Pidon et al., 2020). Moreover, the ever‐increasing availability of sequenced genomes requires more efficient systems for speeding up the identification of functionally important loci (Andolfo et al., 2021a,b). To test the allele mining efficiency of the HRP pipeline, we performed a genome‐wide homolog prediction in five cucurbit genomes using the protein sequence (GenBank accession DQ287965) encoded at the *Fusarium* wilt resistance locus *Fom‐2* isolated from *Cucurbita melo* (Joobeur et al., 2004).

**Figure 3:**
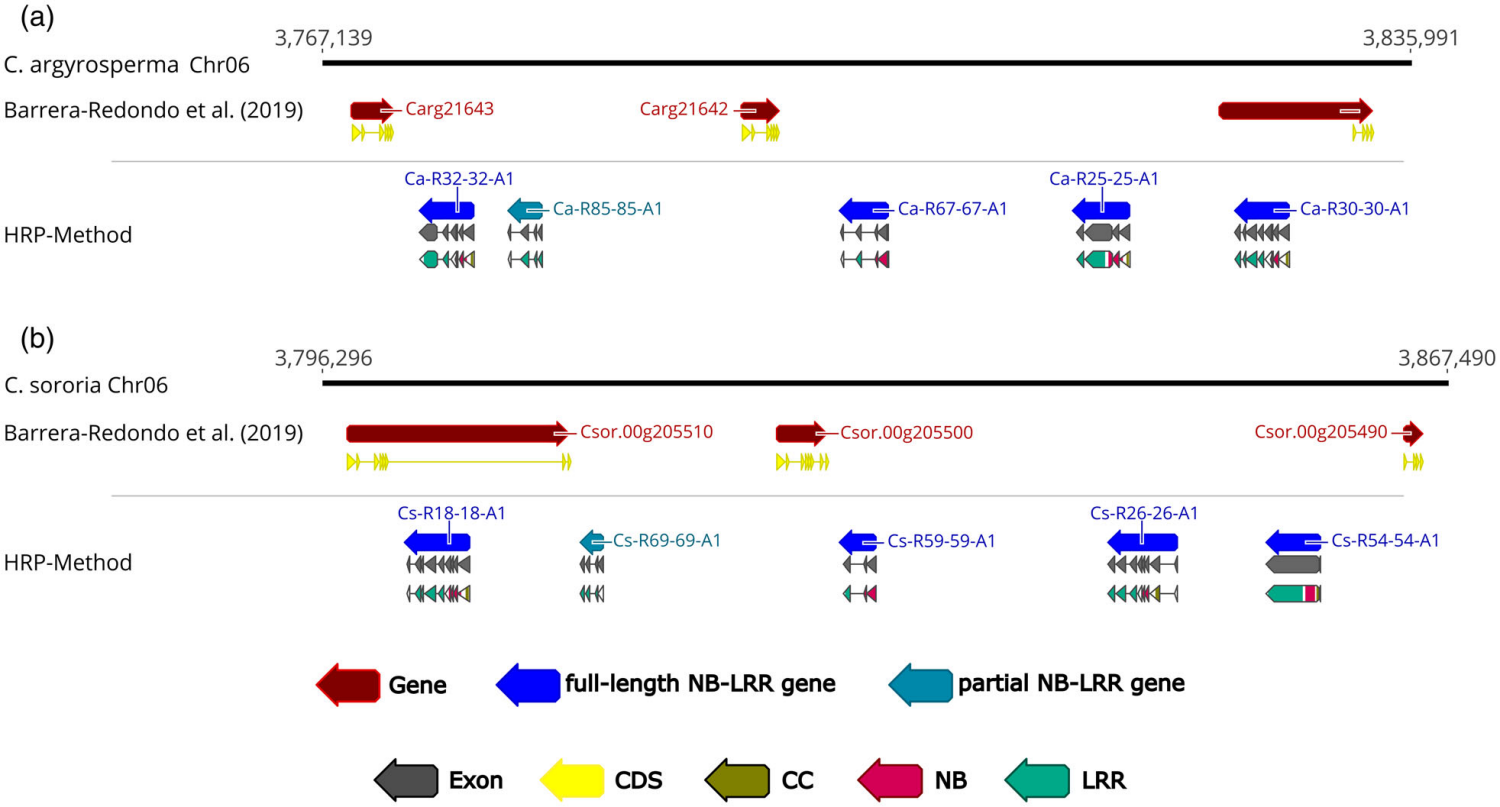
Clusters of *Fom‐2* homologs in *Cucurbita* missed by automatic gene annotation pipelines. NB‐LRR gene clusters composed of four full‐length (blue arrows) and one partial (turquoise arrow) R‐genes predicted by HRP on chromosome 6 of (a) *C. argyrosperma* and (b) its wild relative, *C. sororia*. The *Cucurbita* genome annotation released by Barrera‐Redondo et al. (2019) is depicted as burgundy arrows. The corrected annotation was confirmed in an InterProScan analysis. Domains are shown as gold (CC), rose (NB) and spring green (LRR) arrows. [Colour figure can be viewed at wileyonlinelibrary.com]

Indeed, with a single input sequence HRP found more than triple the number of *Fom‐2* homologs when compared to homologs determined by conventional domain search from an independent study (Andolfo et al., 2021b). A total of 148 full‐length *Fom‐2* homologs were predicted by the HRP method within the genomes of *Cucurbita argyrosperma*, *Cucurbita maxima*, *Cucurbita moschata*, *Cucurbita pepo* and *Cucurbita sororia* (Table S10). In contrast, Andolfo et al. (2021b) identified only 46 full‐length NB‐LRR homologs, which were all confirmed by HRP. No *Fom‐2* locus was included in the *C. argyrosperma* automated gene annotation. Only two and six full‐length *Fom‐2* homologs were predicted in *C. pepo* and *C. sororia* genomes, respectively, from automated annotation pipelines. A substantially higher number of *Fom‐2* homologs were presented in *C. maxima* (12) and *C. moschata* (26) genome annotations (Table 4). Two entirely new gene clusters of *Fom‐2* homologs missed by automatic gene annotation pipelines were uncovered on chromosome 6 of *C. argyrosperma* (Figure 3a) and *C. sororia* (Figure 3b) by HRP. Most likely, the genome annotation pipelines based on public databases for TEs had masked several R‐loci in the *Cucurbita* genomes (Andolfo et al., 2021b).

**Table 4:** Number of full‐length *Fom‐2* homologs identified in five *Cucurbita* genome assemblies using HRP or PDS. All NB‐LRR genes previously annotated by PDS were confirmed by HRP using one *Fom‐2* homolog as input sequence

	Cucurbit genome assemblies				
	*C. argyrosperma*	*C. maxima*	*C. moschata*	*C. pepo*	*C. sororia*
	(v2.0)	(v1.1)	(v1.0)	(v4.1)	(v1.0)
PDS^a^	–	12	26	2	6
HRP	37	14	35	26	36

^a^ Number of *Fom‐2* homologs identified by conventional domain search (Andolfo et al., 2021b).

## DISCUSSION

In the last years, NB‐LRR gene annotation based on automated gene prediction has proven to be inaccurate and incomplete (Andolfo et al., 2014; Jupe et al., 2013). This and the increasing availability of new fully sequenced genomes stimulated us to develop a method that overcomes limitations of the automatic gene annotation processes. In this work, we present a powerful method for full‐length homology‐based R‐gene prediction (HRP) that could be used for the detection of R‐genes for future use in breeding for resistance to plant pathogens. The HRP method permitted an accurate identification of the full tomato NB‐LRR gene repertoire and showed better performance in full‐length R‐gene discovery than the RenSeq approach. Most likely, the motifs‐based annotation derived from a very limited set of NB‐LRR genes (Jupe et al., 2012) and probe libraries using 120‐mer baits to capture target R sequences have not always sufficiently addressed the complex organization of full‐length tomato R‐genes (Andolfo et al., 2014).

Several tools are available to perform R‐gene annotation in plant genomes (Kourelis et al., 2021). However, most of these programs rely on gene sets as input for PDS. Therefore, their performance will always be constrained by the quality of the input gene sets and by the repeat masking approaches used during automated genome annotation (Bayer et al., 2018). For example, when using RGAugury (Li et al., 2016) about 30% of NB‐LRR genes identified by HRP were not detected. Over the last few years, some methods have been developed to work on non‐annotated genomes (Funk et al., 2018; Steuernagel et al., 2020; Toda et al., 2020), but the identified genomic regions containing putative R‐genes need further manual curation to delineate gene structures. HRP is the first pipeline that outputs NB‐LRR gene models, and its accuracy was confirmed by the high overlap of full‐length NB‐LRR gene models annotated by iTAG with those predicted by HRP.

Plant genomes host numerous repeats due to the amplification of diverse TE classes, which can be the major components of large plant genomes (Kim et al., 2014; Wicker et al., 2018). TEs represent the main contributors of intraspecific genomic diversity and one of the major forces driving genome evolution, in that they are subject to rapid turnover (Feschotte et al., 2002). Regrettably, the open reading frames within TEs can confound gene prediction using automated genome annotation pipelines. Indeed, prior to gene prediction, repetitive regions are usually masked by searching for structural features such as TE‐associated domains of known repetitive elements (Bao et al., 2015). Over 20 years ago it was reported that R‐genes are associated with TEs, which helps to diversify the R‐gene repertoire (Richter & Ronald, 2000). In consequence, automated repeat masking approaches have unintentionally masked genes unrelated to transposons leading to a misprediction of R‐genes within genome assemblies of several plant species (Bayer et al., 2018).

Two different repeat masking approaches were used in the automatic genome annotations of RefBv‐1.0 and RefBeet‐1.2 assemblies (Dohm et al., 2014; Minoche et al., 2015). Indeed, the conventional NB‐LRR gene annotation (Andolfo et al., 2021a,b; Meyers et al., 2003), which searches for the typical R‐domains within automatically predicted proteomes, has shown a large difference regarding R‐genes discovered in either sugar beet genome assembly. The automatic gene annotation pipeline proved disappointing even when the genome assembly was derived from long‐read sequencing (Funk et al., 2018; McGrath et al., 2020). In contrast, HRP identified several new R‐genes compared to PDS and predicted a higher number of full‐length NB‐LRR genes also in the PacBio‐based EL10.1 genome assembly (Funk et al., 2018).

Correct and comprehensive description of the R‐gene repertoire in a fully sequenced plant genome is an important step to the discovery of novel NB‐LRR loci with potentially functional importance. Our method is distinguished from standard R‐gene annotation in that it realizes a whole‐genome homology‐based NB‐LRR prediction of full‐length (functional) and partial (non‐functional pseudogenes) gene models. The efficiency of R‐gene prediction using HRP was confirmed by the high number of shared full‐length NB‐LRR genes predicted in two sugar beet genome assemblies.

The NB‐LRR gene family evolves rapidly, as plants must adapt to changing pathogen populations for survival. Tandem duplication of genes, exon shuffling and gene gains and losses are the result of strong natural selection acting on R‐gene loci (Chavan et al., 2015; Seo et al., 2016). Several studies with focus on genome‐wide NB‐LRR gene characterization document the species‐specific diversification of R‐gene repertories (Andolfo et al., 2021a,b; Barchi et al., 2019). An accurate R‐gene identification pipeline always needs to be calibrated on the plant genome to explore. HRP uses genome‐specific gene models to directly interrogate the assembly studied. The accession chosen as the reference genome of a plant species does not contain all the functional copies of R‐genes of that particular species. Indeed, NB‐LRR homologs may have undergone pseudogenization following domestication and breeding (Andolfo et al., 2021a,b). Therefore, it is important to define partial NB‐LRR genes in a reference genome because these genes may have functional alleles in other accessions. HRP has also turned out to be more accurate than the RenSeq approach in the identification of partial NB‐LRR genes (Jupe et al., 2013). The NB‐LRR loci predicted by HRP could be considered *bona fide* gene models, as they are generally based on protein‐encoding gene models supported by experimental evidence (e.g., RNA‐Seq data or protein homology) (Barrera‐Redondo et al., 2019; Barrera‐Redondo et al., 2021; Dohm et al., 2014; Minoche et al., 2015; Sato et al., 2012).

We have also tested our method for allele mining for *Fom‐2*, a functionally characterized R‐gene conferring resistance to *Fusarium oxysporum* f. sp. *melonis* races 0 and 1 in melon (*Cucumis melo*) (Joobeur et al., 2004), in five different *Cucurbita* genome assemblies (Barrera‐Redondo et al., 2019; Barrera‐Redondo et al., 2021; Montero‐Pau et al., 2018; Sun et al., 2017). Interestingly, HRP has proven to be much more efficient than the PDS method in identifying specific R‐gene homologs. Allele mining allows dissecting naturally occurring allelic variation, tracing the evolution of alleles and identification of new haplotypes (Pidon et al., 2020) which has potential applications in crop improvement programs. The HRP method is a more refined ‘mining’ strategy for accelerating the process of allele discovery, extending the search to all the R‐gene homologs present in the genome. R‐gene identification becomes more and more important as the ease of generating high‐quality genome assemblies from long‐read data increases. The HRP pipeline can be used to identify and isolate novel R‐genes from crop gene pools to suitably deploy them for the development of improved cultivars.

## EXPERIMENTAL PROCEDURES

### Genome assemblies and gene sets

To validate the full‐length HRP pipeline we used the *S. lycopersicum* Heinz‐1706 genome (v2.4) and its NB‐LRR gene annotations as reported by Andolfo et al. (2013, 2014). The tomato genome sequence (S_lycopersicum_chromosomes.2.40.fa), gene model annotation (ITAG2.3_gene_models.gff3) and protein sequences (ITAG2.3_proteins.fa) were downloaded from the FTP site of SGN (https://solgenomics.net/). We also used two genome assemblies (RefBv‐1.0 and RefBeet‐1.2) of the double haploid sugar beet genotype KWS2320 (Dohm et al., 2014) and another sugar beet assembly (EL10) derived from an inbred derivative of sugar beet line C869 (Funk et al., 2018; McGrath et al., 2020), as well as five *Cucurbita* genome assemblies (*C. argyrosperma*, *C. maxima*, *C. moschata*, *C. pepo* and *C. sororia*) for comparative purposes (Barrera‐Redondo et al., 2019; Barrera‐Redondo et al., 2021; Montero‐Pau et al., 2018; Sun et al., 2017). *Beta* sp. and spinach genomic data and/or gene sets were obtained from the *Beta vulgaris* Resource database (https://bvseq.boku.ac.at/), sugar beet EL10 data were downloaded from CoGe (https://genomevolution.org/coge/GenomeInfo.pl?gid=54615) and *Cucurbita* sp. genome sequences and annotated genes were retrieved from CuGenDB (http://cucurbitgenomics.org/). The melon Fom‐2 protein sequence was obtained from GenBank (accession number DQ287965).

### Pipeline for full‐length homology‐based NB‐LRR gene identification

**Figure 4:**
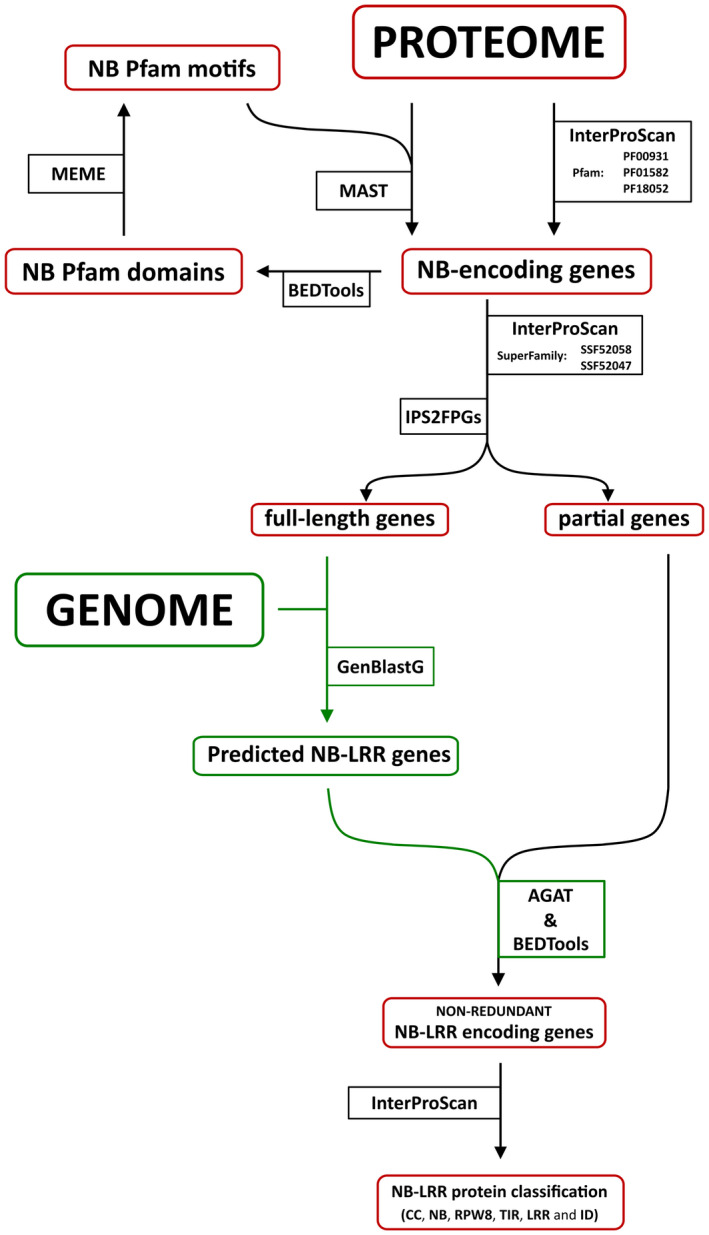
Step‐by‐step workflow for NB‐LRR gene prediction and annotation. The HRP pipeline was designed to detect conserved domains (CC: PF18052; LRR: SSF52058 and SSF52047; NB: PF00931; TIR: PF01582) found in well‐characterized plant R‐genes by integrating results generated from protein motif/domain‐based search (MEME/MAST and InterProScan software), combined with an R‐gene prediction based on homology to full‐length NB‐LRR genes by GenBlastG within assembled genome sequences. [Colour figure can be viewed at wileyonlinelibrary.com]

The pipeline uses a double‐pass process for NB‐LRR gene identification directly in the genome, based on the *de novo* prediction of R‐loci and using the full‐length NB‐LRR genes that were automatically annotated in the studied taxa as R‐gene models (Figure 4).

The first pass focuses on the identification of full‐length NB‐LRRs in the protein‐encoding gene model dataset of automated genome annotation using a conventional R‐protein domain search (PDS). InterProScan v5 (Jones et al., 2014) software is used to search for the Pfam domains NB (PF00931), Rx‐N (PF18052) and TIR (PF01582) using default settings. During the first pass, the NB Pfam domain sequences (PF00931) of full‐length NB‐LRR genes identified by InterProScan were decomposed into motifs using Multiple EM for Motif Elicitation (MEME) software with default settings except for the following parameters ‐nmotifs 19 ‐minw 4 ‐maxw 7 (Bailey et al., 2006). The NB Pfam domain sequences were retrieved using getfasta implemented in BEDTool (v2.30.0) (Quinlan & Hall, 2010), as it is the most conserved region present in all R‐genes of green plants (Andolfo et al., 2019). The identified MEME motifs (Table S11) were used for analysis using the Motif Alignment and Search Tool (MAST) with default settings (Bailey et al., 2006) to discover additional NB‐LRR genes that had remained undetected by the foregoing annotation (black in Figure 4).

The second pass of NB‐LRR gene identification is carried out using the homology‐based predictor GenBlastG (She et al., 2011). Full‐length NB‐LRR protein sequences, identified in the first step described above, were used as queries for whole‐genome NB‐LRR prediction using default settings (green in Figure 4). The alignment scores for each aligned pair of amino acids were based on the BLOSUM62 score matrix. The analyses were carried out using the default cut‐off e‐value of 0.01 for statistical confidence. We removed predicted gene models that spanned a genomic region longer than 20 kb using AGAT (v0.7.0) (Dainat, 2021). Overlapping NB‐LRR gene models predicted by GenBlastG were removed using clusterBed (−s) implemented in BEDTool (v2.30.0) (Quinlan & Hall, 2010). The NB‐LRR gene models encoding the longest putative NB‐LRR protein were retained. Finally, the protein domain architecture of predicted NB‐LRR proteins was annotated using the programs Coils, Pfam and SuperFamily as implemented in InterProScan v5 using default parameters (Jones et al., 2014). Pseudogenes with a complete R‐protein domain architecture (CC or RPW8 or TIR in combination with NB and LRR) were also identified by the pipeline. The HRP pipeline can be run using the instructions deposited in the GitHub public repository (https://github.com/AndolfoG/HRP.git).

### Comparison of HRP and PDS methods

The candidate R‐loci identified from HRP and PDS methods were exported in GFF3 format and imported into the IGV genome browser (Thorvaldsdóttir et al., 2013) for comparison and visual inspection. The full‐length NB‐LRR genes identified in RefBv‐1.0 and RefBeet‐1.2 genome assemblies were analyzed using InParanoid v5 (Jones et al., 2014) with default parameters. We used a confidence score threshold of 1 to directly estimate gene–gene correspondences between the HRP and PDS‐only predicted proteins. The comparison of gene models predicted by HRP and by iTAG was performed using bedtools intersect (BEDTools v2.30.0) (Quinlan & Hall, 2010). The tomato NB‐LRR protein annotation was performed by RGAugury (Li et al., 2016) on the *S. lycopersicum* Heinz‐1706 proteome (v2.3) using the default cut‐off e‐value of 1e−5 for statistical confidence.

## CONFLICT OF INTEREST

The authors declare no conflicts of interest.

## AUTHOR CONTRIBUTIONS

GA, HH and JCD conceived the study, GA designed experiments and analyzed the data. GA wrote the first manuscript draft. All authors finalized the manuscript draft. All authors approved the final manuscript.

## Supporting information


**Table S1.** Full‐length NB‐LRR proteins identified by Andolfo et al. (2013) used to re‐annotate tomato R‐genes. C: coiled‐coil; L: leucine‐rich repeat; N: nucleotide binding; R: resistance to powdery mildew 8; T: Toll/interleukin receptor.
**Table S2.** Information for 363 NB‐LRR genes annotated in the tomato genome (Heinz 1706 cultivar) using the HRP method, including strand information, start and end positions on the chromosome and the protein class.
**Table S3.** List of 103 (out of 105) tomato NB‐LRR genes predicted by HRP, previously identified by RenSeq analysis. Columns ‘Gene IDs’, ‘Expression status’ and ‘Description of Annotation’ refer to the results as presented by Andolfo et al. (2014). Column ‘Expression status’: E: expressed; NE: not expressed.
**Table S4.** Information for 48 full‐length NB‐LRR gene models predicted by HRP in the tomato genome (Heinz 1706 cultivar) and confirmed by iTAG, including strand information and start and end positions on the chromosome.
**Table S5.** List of R‐proteins annotated by RGAugury in the tomato proteome (ITAG2.3). C: coiled‐coil; L: leucine‐rich repeat; N: nucleotide binding; T: Toll/interleukin receptor.
**Table S6.** Full‐length NB‐LRR proteins identified using protein motif/domain‐based search (PDS) within published gene sets of wild and cultivated beets and spinach. These genes were used as input for HRP‐based prediction of R‐genes within the RefBv‐1.0, RefBeet‐1.2 and EL10 sugar beet genome assemblies. C: coiled‐coil; L: leucine‐rich repeat; N: nucleotide binding; R: resistance to powdery mildew 8; T: Toll/interleukin receptor.
**Table S7.** List of NB‐LRR genes predicted and annotated in the RefBv‐1.0 and RefBeet‐1.2 genome assemblies. C: coiled‐coil; L: leucine‐rich repeat; N: nucleotide binding; R: resistance to powdery mildew 8; T: Toll/interleukin receptor.
**Table S8.** Number of NB‐LRR homolog groups identified by InParanoid in the RefBv‐1.0 and RefBeet‐1.2 genome assemblies.
**Table S9.** List of NB‐LRR genes predicted by HRP in the sugar beet EL10.1 genome assembly.
**Table S10.** Full‐length *Fom‐2* homologs identified in five *Cucurbita* genome assemblies by HRP. C: coiled‐coil; L: leucine‐rich repeat; N: nucleotide binding.
**Table S11.** MEME motifs identified within NB Pfam domains of full‐length NB‐LRR proteins annotated in 10 Caryophyllales proteomes.

## Data Availability

Information on how to run the HRP pipeline is available on GitHub (https://github.com/AndolfoG/HRP.git). Information on input genes and gene predictions can be found at the Beta vulgaris Resource web page (https://bvseq.boku.ac.at/) and in the online supplementary material which is part of this article.
